# Determinants of Return to Work in Kidney Transplant Recipients—A Study from the West Pomeranian Region of Poland

**DOI:** 10.3390/jcm14238549

**Published:** 2025-12-02

**Authors:** Maria Piątak, Sylwia Wieder-Huszla, Joanna Owsianowska, Anna Jurczak, Tomasz Śluzar, Marek Myślak

**Affiliations:** 1 Department of Specialized Nursing, Pomeranian Medical University in Szczecin, 71-210 Szczecin, Poland; 2 Department of General Surgery and Transplantation, Independent Public Voivodship Hospital, 71-455 Szczecin, Poland; 3 Department of Social Medicine and Public Health, Pomeranian Medical University in Szczecin, 71-210 Szczecin, Poland; marek.myslak@pum.edu.pl

**Keywords:** kidney transplantation, return to work, employment, quality of life, sociodemographic factors, renal replacement therapy

## Abstract

**Background:** Kidney transplantation enables not only longer survival but also reintegration into professional and social life. Understanding the determinants of post-transplant employment is crucial for optimizing rehabilitation programs. **Methods:** A cross-sectional study was conducted between 2019 and 2021 among 94 kidney transplant recipients treated in two outpatient clinics. Data were collected using a structured interview and medical record analysis. Sociodemographic and clinical variables were assessed. Statistical analysis included chi-square tests, Spearman’s correlation, and logistic regression. All variables that demonstrated statistical significance in the univariate analysis were entered into the multivariate logistic regression model to identify independent predictors of post-transplant employment. A *p*-value < 0.05 was considered significant. **Results:** Prior to transplantation, 67.0% of patients were professionally inactive. After transplantation, 66.0% resumed employment, with 41.9% returning to work within six months. Return to work was positively correlated with higher education (*p* < 0.009), good financial status (*p* < 0.040), and longer time since transplantation (*p* < 0.001). Employment prior to transplantation, donor type, and duration of dialysis also significantly influenced outcomes. In the multivariate logistic regression, only marital status and place of residence remained independent predictors of return to work. **Conclusions:** More than half of transplant recipients successfully returned to work. Sociodemographic (education, marital status, financial situation, residence) and medical factors (dialysis duration, donor type, time since transplantation, prior employment) determined vocational reintegration. Further multicenter, longitudinal studies are needed to identify modifiable factors and design interventions enhancing post-transplant employability.

## 1. Introduction

Kidney transplantation is one of the most effective treatment modalities for end-stage renal disease, offering not only prolonged survival but also improvements in both physical and mental health, and the opportunity to return to social and professional activities [1]. The primary goal of transplantation is to enable individuals to lead fuller and more satisfying lives. Effective rehabilitation and appropriate post-transplant care support social reintegration, although patients engaged in physically demanding jobs often encounter limitations, whereas those in less physically intensive occupations usually demonstrate stable work functioning [2]. Return to work after kidney transplantation is a complex and multifactorial process that requires adaptation to new health and occupational circumstances. For many individuals, employment constitutes the main source of income; however, it also provides self-esteem, a sense of social belonging, and personal development opportunities. Professional activity contributes to improved quality of life and enhances social participation. At the same time, some patients perceive themselves as disabled and fear complications or graft rejection, which may affect not only their career decisions but also their social interactions. Despite the importance of employment outcomes, knowledge on work capacity, absenteeism, and productivity after solid-organ transplantation remains limited, even though these indicators are crucial for long-term social and vocational reintegration [3]. Multiple factors have been identified as determinants of RTW, including clinical parameters (underlying disease, treatment modalities before transplantation, duration of dialysis, donor type, comorbidities) and sociodemographic variables (age, education, financial status, place of residence). Social and family support also plays a pivotal role in facilitating professional reintegration. Findings by Knobbe et al. emphasize that kidney transplant recipients are able to perform well in the workplace, and illness does not necessarily constitute a barrier to productivity. These observations are important for reducing stigma associated with employment of transplant recipients [2]. RTW rates, however, vary considerably across countries and centers. A nationwide registry-based study from Denmark (2005–2019) demonstrated that employment among kidney transplant recipients remained significantly lower compared to the general population throughout the observation period, both before and after transplantation. Although an increase in work participation was observed one year after transplantation, this improvement was only partial and did not exceed general population trends [4]. Recent findings by Visser et al. (2022) showed that patients undergoing preemptive kidney transplantation, particularly from living donors, had higher chances of employment and greater job stability compared to those who underwent transplantation after long-term dialysis [3]. In addition, psychosocial factors such as mental health, social support, and economic stability, as well as adherence to post-transplant recommendations, have been increasingly recognized as critical for sustained employment. A 2025 systematic review demonstrated that socioeconomic status—including education, employment history, and social networks—is strongly associated with adherence to immunosuppressive therapy, which indirectly contributes to health stability and employability [5]. Swiss and German studies further suggest that structured rehabilitation programs and vocational counseling can significantly improve reintegration into the labor market within the first year after transplantation [6]. In Poland, there is still a lack of up-to-date, multicenter studies that combine both clinical and psychosocial determinants of post-transplant employment. Existing studies are limited to single-center cohorts and provide only fragmentary insights. Therefore, further research is warranted to better understand the determinants of occupational reintegration and to develop strategies that support successful return to work among kidney transplant recipients.

## 2. Materials and Methods

### 2.1. Study Design and Setting

This observational study was conducted between 1 April 2019 and 30 September 2021 in two transplant outpatient clinics in Szczecin: the Transplant Outpatient Clinic of the Independent Public Clinical Hospital No. 2 of the Pomeranian Medical University and the Transplant Outpatient Clinic of the Independent Public Regional Hospital. The study included all adult kidney transplant recipients followed at the two transplant outpatient clinics in Szczecin, which serve as the only post-transplant care centers in the West Pomeranian region of Poland. There is no other kidney transplant center in this region.

During the study period (1 April 2019–30 September 2021), approximately 50–60 kidney transplantations were performed in both centers combined. This relatively low number reflects the impact of the COVID-19 pandemic, during which transplant activity was temporarily reduced. The present study included patients who had undergone kidney transplantation within the previous 10 years.

A total of 112 kidney transplant recipients were assessed for eligibility during the study period. After applying inclusion and exclusion criteria, 94 participants were enrolled in the final analysis.

The inclusion criteria were:

(1) age ≥ 18 years;
(2) provision of written informed consent;
(3) no need for dialysis treatment after transplantation.

The exclusion criteria included:

(1) refusal to participate (n = 11);
(2) severe mental disorders or cognitive impairment preventing questionnaire completion (n = 7).

**Figure 1:**
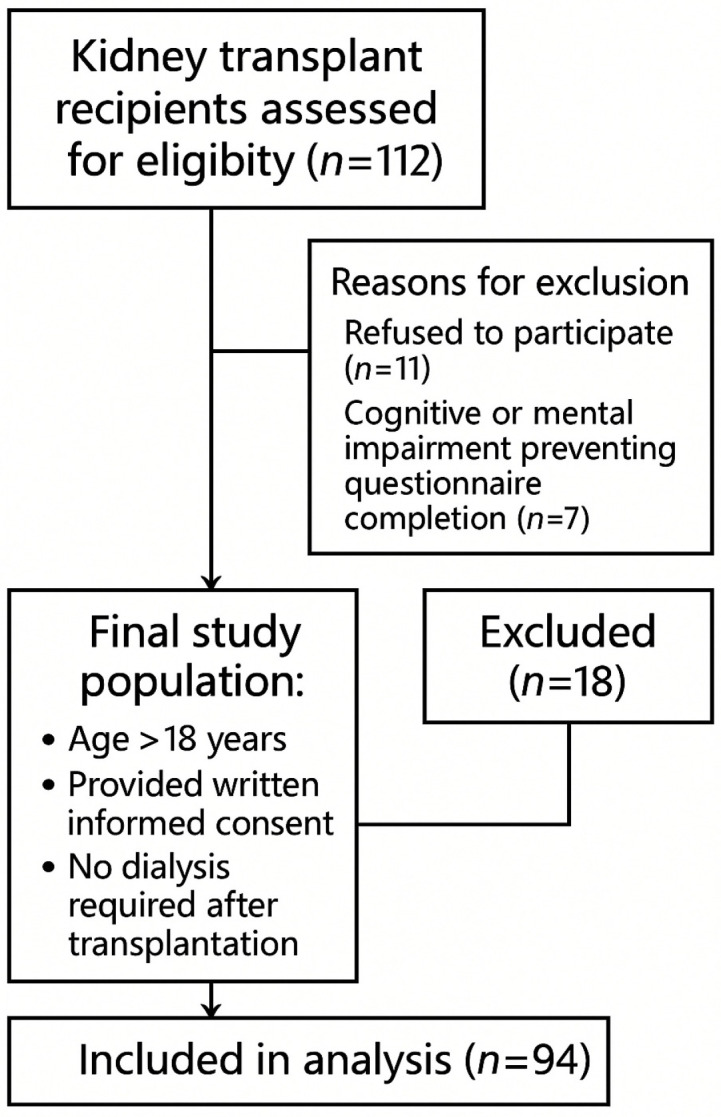
Flow diagram of patient selection.

A detailed flow diagram illustrating patient selection, exclusions, and final sample size is presented in Figure 1.

Patients requiring chronic dialysis after transplantation were excluded. Those who experienced transient delayed graft function (DGF) but recovered kidney function were included.

Of 112 kidney transplant recipients assessed for eligibility, 18 were excluded (11 refused participation, 7 due to cognitive impairment), resulting in a final study group of 94 participants.

The study was conducted in accordance with the Declaration of Helsinki, and the protocol was approved by the Bioethics Committee of the Pomeranian Medical University (approval no. KB-0012/88/19). All data were collected anonymously and confidentially, without the participation of accompanying persons.

### 2.2. Research Instruments

All patients received maintenance immunosuppressive therapy consisting of a calcineurin inhibitor (tacrolimus or cyclosporine), mycophenolate mofetil, and corticosteroids.

The following research instruments were applied:

Self-designed questionnaire (25 items), developed specifically for this study, as no standardized tool was available to comprehensively capture the unique situation of kidney transplant recipients in terms of sociodemographic, clinical, and social-professional functioning. The questionnaire included:

Sociodemographic variables: age, sex, marital status, place of residence, level of education, housing conditions, financial situation, occupational status before and after transplantation;

Clinical variables: cause of end-stage renal disease, type and duration of renal replacement therapy, time on the National organ transplant waiting list, waiting time for transplantation, donor type (living/deceased), number of transplantations, length of hospital stay after transplantation, number of rehospitalizations;

Post-transplant functioning: self-reported health status and well-being, sexual activity before and after transplantation, occurrence of post-transplant complications, adherence to medical recommendations (follow-up visits, immunosuppressive medication, vaccinations, self-care);

Social functioning: family and social relationships, participation in patient associations, cultural activity, sports and recreational activity, hobbies, travel, continuation of education, changes in marital status, and parenthood after transplantation.

Structured interview, conducted during follow-up visits, complementing questionnaire data.

Medical record review and analysis of electronic hospital databases, providing information on treatment course, hospitalizations, and clinical parameters (e.g., creatinine concentration, estimated glomerular filtration rate [eGFR]).

### 2.3. Data Collection

The questionnaires were completed by patients during routine outpatient visits. Written informed consent was obtained from all participants. Questionnaire data were subsequently verified and supplemented with information retrieved from medical documentation and hospital electronic records.

### 2.4. Statistical Analysis

Data analysis was performed using the Statistica software package (Version 14.1.0., StatSoft, Poland). The following statistical methods were applied:

- Chi-square test of independence,

McNemar test for paired comparisons of employment status before and after transplantation,
Spearman’s rank correlation coefficient,
Mann–Whitney U test for non-parametric comparisons.

The normality of continuous variables was verified using the Shapiro–Wilk test. Non-parametric tests (Spearman’s correlation and Mann–Whitney U test) were applied, as most variables did not follow a normal distribution.

Logistic regression was performed to identify the influence of sociodemographic and clinical variables on post-transplant employment.

The logistic regression analysis included all variables that showed statistical significance (*p* < 0.05) in the univariate tests (chi-square or Spearman’s correlation). No additional predefined variables were added.

The results were considered statistically significant at a *p*-value of <0.05.

## 3. Results

### Characteristics of the Participants

The study group consisted of 48 women (51.1%) and 46 men (48.9%), aged between 22 and 63 years, with a mean age of 40.6 ± 8.6 years. The vast majority of the participants were married (58.5%), had secondary (40.9%) or higher education (28.0%), lived in large urban areas (33.0%), and reported good housing conditions (76.6%) and good financial standing (56.4%). In a significant proportion of the patients (67.1%), glomerulonephritis was the primary reason for kidney transplantation. A total of 72.3% of the respondents had undergone renal replacement therapy in the form of hemodialysis prior to transplantation, with 44.7% receiving treatment for no longer than three years. Kidneys from deceased donors were received by 85.1% of the participants, and for most (77.7%) it was their first transplant.

The mean serum creatinine concentration was 1.32 ± 0.45 mg/dL, and the mean estimated glomerular filtration rate (eGFR) was 59.4 ± 18.2 mL/min/1.73 m^2^. Detailed sociodemographic and medical characteristics are presented in Table 1.

Before transplantation, as many as 67.0% of the patients were professionally inactive, reflecting limitations related to their health status. After transplantation, 66.0% of the participants returned to work, although physical labor accounted for only a marginal type of employment (8%). Within the first six months after the surgery, 41.9% of the patients had resumed work, which may indicate a dynamic rehabilitation process and gradual improvement in quality of life. The analysis showed that return to occupational activity was correlated with higher education level (*p* < 0.009), good financial situation (*p* = 0.040), and longer time elapsed since transplantation (*p* < 0.001), as shown in Table 2.

Analysis of other variables revealed that individuals employed before transplantation significantly more often returned to work (*p* < 0.001). Recipients of kidneys from living donors and those with a shorter duration of renal replacement therapy were also more likely to return to work (donor type: *p* = 0.021; duration of RRT: *p* = 0.039), as shown in Table 3.

In-depth analysis revealed that residents of large cities with populations over 100,000 had significantly higher chances of returning to work compared to patients living in rural areas (*p* < 0.05). Conversely, divorced individuals had lower chances of employment than those who were single (*p* < 0.012) (Table 4).

## 4. Discussion

Returning to work after kidney transplantation is one of the goals of comprehensive rehabilitation, bringing tangible benefits to both the patient and society. Chronically ill patients frequently face challenges in obtaining and maintaining employment. Due to the nature of their condition, they often receive little support or understanding from employers and colleagues, or are forced to accept jobs that do not match their education or qualifications. Fear of employers’ reactions and the desire to avoid being perceived as disabled often lead to reluctance to disclose their chronic illness. Such concerns and challenges may result in feelings of social exclusion, while the lack of acceptance in the workplace can negatively affect patients’ self-esteem [7,8]. Nagasawa et al.’s research revealed that some patients opted for nocturnal dialysis in order to keep their condition hidden from their employer [7].

Despite numerous challenges, some dialysis patients choose to engage in professional activity, as such involvement has a significant impact on quality of life, which is closely linked to health status and contributes to one’s perception of that status [9]. Engaging in employment not only improves patients’ subjective assessment of their health but also serves as an important foundation for maintaining self-esteem. Employment not only provides a source of income but also shapes lifestyle choices and facilitates access to a broad range of goods and services, including quality education and specialized healthcare. Returning to work after kidney transplantation is an important step in the process of social reintegration, fostering the restoration of both financial and psychological stability.

Available data indicate that, despite improvements in health and physical condition following transplantation, a significant proportion of patients do not return to work [10]. According to Strugała et al., as many as 87.0% of dialysis patients remain unemployed, primarily due to health-related reasons or the need to receive a disability pension [3]. In the present study, 66.0% of respondents resumed professional activity after kidney transplantation, with 41.9% returning to work within the first six months after surgery. Most participants took up white-collar or mixed-type jobs, which is understandable given the greater flexibility and lower physical demands compared to occupations requiring intense physical effort. Similar results were reported in studies conducted among comparable patient groups in Poland, the Czech Republic, and Germany, where over 60.0% of post-transplant patients returned to work in Poland and the Czech Republic, while in Germany it was approximately 50.0% [11]. However, a study by Ostrowski’s team showed that more than 63.0% of working transplant recipients were employed part-time [12]. Post-transplant employment rates in the United States have been reported at around 43–44.0% [13], while in Switzerland and Japan the figures were 56.0% and 76.0%, respectively [14,15].

Numerous researchers have attempted to identify the factors that predispose kidney transplant recipients to return to work. Danuser et al. found that the most significant determinants included pre-transplant employment, younger age, and educational attainment [16]. Similarly, studies by Eppenberger and Nour identified a shorter duration of dialysis prior to transplantation, receipt of a kidney from a living donor, and preemptive transplantation as key predictors of post-transplant employment [17,18]. According to Mouelhi et al., kidney transplants from living donors are often associated with better and more stable long-term quality of life and a lower risk of graft rejection [15]. These patients generally have more favorable conditions for returning to work after transplantation. Our findings demonstrated that return to employment was significantly correlated with education level, financial status, duration of renal replacement therapy, time elapsed since transplantation, and donor type. Research conducted by Visser et al. showed that patients who received a kidney from a living donor—particularly in preemptive procedures—were significantly more likely to resume work following transplantation. Other factors strongly associated with higher levels of occupational activity included younger age and the absence of comorbidities [14]. A study by Miyake’s team reported that 85% of patients returned to work within 12 months from kidney transplantation, with 76% employed full-time. Data analysis also revealed higher return-to-work rates among those in managerial positions, which the authors attributed to greater job flexibility and the ability to adapt professional responsibilities to the patient’s health status [19]. In a literature review conducted by D’Egidio et al., it was emphasized that returning to work after kidney transplantation is a complex and multifactorial process, influenced not only by medical condition but also by sociodemographic and psychosocial factors [3].

The study by Sahota et al. demonstrated that a prolonged period of disability prior to transplantation negatively affects post-transplant occupational activity [20]. This finding appears to be supported by the results of the present study, as the participants who had been employed before transplantation were more likely to return to work. It was also observed that living in large urban areas was associated with a higher likelihood of successful vocational reintegration.

Although hospitalization data and patients’ motivation to return to work were not analyzed in detail, these aspects may also influence post-transplant occupational outcomes and should be considered in future studies.

In Poland, dialysis patients are eligible for disability pensions and partial reimbursement of transportation costs during renal replacement therapy. These benefits are discontinued after successful transplantation if the patient resumes employment. This social context may influence the decision to return to work; however, detailed information on benefit status was not collected in this study and represents a limitation.

Despite the obvious benefits, kidney transplant recipients still face challenges such as a lack of full social understanding of their health situation and the specific needs arising from their disease course. In summary, the effectiveness of professional reintegration after kidney transplantation depends on multiple factors. Inclusion in active life not only helps these individuals but also brings social benefits. There remains a need for greater societal awareness of the challenges faced by chronically ill patients. Ultimately, promoting occupational and social activity should be an integral part of every transplant program.

## 5. Conclusions

This study demonstrated that more than half of kidney transplant recipients were able to resume professional employment, with education level, financial standing, marital status, place of residence, duration of renal replacement therapy, time elapsed since transplantation, donor type, and employment prior to transplantation emerging as key determinants of return to work. An innovative aspect of this study is the inclusion of both clinical and psychosocial variables, which provides a broader perspective on occupational reintegration after transplantation.

However, several limitations should be acknowledged. The study was conducted in a single region (Szczecin), which may limit the generalizability of the findings to other populations. The sample size was relatively modest, and self-reported data could have introduced response bias. Moreover, the cross-sectional design precludes establishing causal relationships between the analyzed factors and employment outcomes.

Future research should therefore focus on multicenter, prospective studies with larger cohorts, incorporating standardized measures of psychosocial functioning, work ability, and quality of life. Such studies would allow for more robust conclusions and the development of tailored interventions to support kidney transplant recipients in achieving stable occupational reintegration.

## Figures and Tables

**Table 1:** Sociodemographic and clinical characteristics of kidney transplant recipients (*n* = 94).

Sociodemographic Data		
**Marital status**	***n***	**%**
Single	33	35.1
Married	55	58.5
Divorced	6	6.4
**Education**	***n***	**%**
Primary	2	2.2
Vocational	21	22.6
Secondary	38	40.9
Higher	6	6.5
Professional higher	26	28.0
**Place of residence**	***n***	**%**
Rural areas	25	26.6
Urban areas (≤20,000 residents)	18	19.1
Urban areas (≤100,000 residents)	20	21.3
Urban areas (>100,000 residents)	31	33.0
**Housing situation**	***n***	**%**
Average	22	23.4
Good	72	76.6
**Financial situation**	***n***	**%**
Insufficient	6	6.4
Average	35	37.2
Good	53	56.4
**Medical data**		
**Cause of kidney disease**	***n***	**%**
Diabetes	4	4.3
Glomerulonephritis	58	61.7
Polycystic kidney disease	2	2.1
Hypertensive nephropathy	4	4.3
Other	17	18.1
Unknown	9	9.6
**Renal replacement therapy before kidney transplantation**	***n***	**%**
Not applicable	5	5.3
Peritoneal dialysis	21	22.3
Hemodialysis	68	72.3
**Duration of renal replacement therapy**	***n***	**%**
Pre-dialysis	5	5.3
Up to 36 months	42	44.7
Over 36 months	36	38.3
No data	11	11.7
**Waiting time for transplantation**	***n***	**%**
Up to 36 months	72	76.6
Over 36 months	14	14.9
No data	5	5.3
Not applicable	3	3.1
**Donor Type**	***n***	**%**
Living	14	14.9
Deceased	80	85.1
**Number of Transplants**	***n***	**%**
1	73	77.7
2	14	14.9
3	6	6.4
4	1	1.1

**Table 2:** Associations between sociodemographic factors and return to work after kidney transplantation.

Variable	r	*p*-Value
Education	0.27	0.009
Financial standing	0.21	0.040
Time of return to work	0.65	*p* < 0.001

Chi-square and Mann–Whitney U tests were applied. Statistically significant results (*p* < 0.05).

**Table 3:** Associations between medical factors and return to work after kidney transplantation.

Variable	*p* (chi^2^)
Sex	0.847
Age	0.235
Treatment method	0.496
Number of transplantations	0.971
Donor type	0.021
Duration of dialysis therapy	0.039
Employment before transplantation	*p* < 0.001

chi^2^—chi-square test of independence.

**Table 4:** Impact of variables on employment after transplantation (logistic regression analysis).

Variable	OR	95% CI (Lower)	95% CI (Upper)	*p*-Value
Marital status (married vs. Single)	0.065	0.008	0.552	0.012
Place of residence (city ≤ 100,000 vs. rural area)	8.941	1.011	79.052	0.048

OR—odds ratio, *p*—significance level.

## Data Availability

All data are contained in this article.

## Abbreviations

The following abbreviations are used in this manuscript:

eGFR	estimated glomerular filtration rate
RTW	return to work
RRT	renal replacement therapy
OR	odds ratio
CI	confidence interval
*p*	significance level
